# Long-term effects of the COVID-19 lockdown on the structural and functional outcomes of neovascular AMD patients in Suzhou, China

**DOI:** 10.1371/journal.pone.0319677

**Published:** 2025-03-20

**Authors:** Zheyao Gu, Xiangying Luo, Ruizhu Sun, Ting Xi, Chunyuan Zhang

**Affiliations:** Department of Ophthalmology, The Affiliated Suzhou Hospital of Nanjing Medical University, Suzhou Municipal Hospital, Suzhou, China; UCMI: University College MAIWP International, MALAYSIA

## Abstract

**Background:**

Timely anti-vascular endothelial growth factor (VEGF) therapy is essential for visual function in neovascular age-related macular degeneration (nAMD). The coronavirus pandemic has led to unprecedented delays in anti-VEGF intravitreal therapy because of the need to reduce hospital attendance.

**Objectives:**

To assess the long-term impact of COVID-19 pandemic-related delays in intravitreal anti-VEGF therapy on nAMD patients.

**Methods:**

This was a retrospective study of 98 patients (102 eyes) with nAMD whose anti-VEGF treatments were interrupted for >  8 weeks due to the COVID-19 pandemic. Best-corrected visual acuity (BCVA), central retinal thickness (CRT) and anatomical characteristics on spectral domain optical coherence tomography (SD-OCT) were measured at baseline, at the last follow-up visit before treatment interruption (V0), at the first visit after the COVID-19 lockdown had ended (V1), at the six-month follow-up (V-6 months) and at the final visit at the 1-year follow-up (V-final). The control group included nAMD patients who had completed at least three anti-VEGF treatments and received consecutive follow-up with timely anti-VEGF treatments for one year.

**Results:**

After one year of regular follow-up and standardized treatment, the treatment-interrupted group (TIG) had significantly worse visual acuity than the treatment-continuous group (TCG) (0.71 ± 0.38 vs. 0.52 ± 0.32, p < 0.001); however, there was no significant difference between the groups in the mean CRT (273.95 ± 112.96 µm vs. 261.43 ± 90.66 µm, p > 0.05). Furthermore, subgroup analysis revealed that, compared with those before treatment interruption, the BCVA of the TIG patients slightly improved, but the mean CRT and related activity indices returned to baseline values according to OCT imaging (all p > 0.05). Multiple linear regression analysis revealed that longer treatment interruption was associated with greater deterioration in visual acuity (p = 0.009).

**Conclusion:**

Treatment interruption for more than 8 weeks had a sustained negative impact on visual acuity in treated eyes one year later. For nAMD patients, continuous treatment, regardless of the underlying regimen, remains critical.

## 1. Introduction

On 11 March 2020, coronavirus disease 2019 (COVID-19) was designated a global pandemic, and as of 1 February 2022, the disease had infected more than 370 million people and caused 5.6 million deaths globally [1]. To curb the spread of severe acute respiratory syndrome coronavirus 2 (SARS-CoV-2), the government of Suzhou, China, implemented a strict public health policy for the city from 15 February to 5 May 2022, including restrictions on hospital activities, social distancing rules and a city-wide lockdown. Among these measures, access to eye disease screening and surgery was greatly restricted.

Age-related macular degeneration (AMD) is a degenerative retinal disease that causes progressive and irreversible loss of central vision and is the primary cause of blindness in people older than 50 years, seriously impacting patients’ quality of life [2]. The main pathological features of neovascular AMD (nAMD) are macular neovascularization (MNV), intraretinal or subretinal fluid accumulation, hemorrhage and retinal pigment epithelial detachment (PED) [3]. Notable clinical characteristics include rapid visual distortion and loss of central vision over a period of weeks to months. The pathogenesis of nAMD has not been elucidated but may be related to retinal pigment epithelium (RPE) damage, mitochondrial dysfunction, oxidative stress, inflammation, and activation of the complement pathway [4]. Despite the current advances in the treatment of this disease, nAMD still cannot be cured or reversed.

Currently, intravitreal injections of anti-vascular endothelial growth factor (VEGF) drugs such as ranibizumab and aflibercept are widely used as first-line treatments for patients with nAMD [5,6]. Regular anti-VEGF therapy, whether via a treatment-and-extend regimen or pro re nata, is essential for maximizing visual improvement in these patients. Owing to factors such as the hospital lockdown caused by COVID-19 and the increased psychological burden on patients, the use of the anti-VEGF regimen for many patients with nAMD has been delayed or cancelled.

Previous studies have reported that the best-corrected visual acuity (BCVA) decreases to varying degrees in nAMD patients whose injections were delayed due to lockdowns related to COVID-19 [7–12]. However, the results of recent research on changes in visual acuity and anatomical status in nAMD patients, as well as analyses of relevant factors affecting patient outcomes, are still controversial [7,13–17].

The aim of this study was to evaluate the changes in visual functional and anatomical outcomes of nAMD patients who experienced at least an 8-week treatment delay caused by COVID-19 and who were followed up after resuming anti-VEGF treatment during a year of regular treatment and to elucidate the reasons for these changes. For this purpose, we examined changes in best-corrected visual acuity (BCVA), central retinal thickness (CRT), and anatomical features and analyzed whether these changes were associated with worsening visual acuity.

## 2. Methods

### 2.1. Ethics statements

The Ethics Committee of Suzhou Municipal Hospital approved this study (Project No. KL901177), which adhered to the principles of the Helsinki Declaration. Before receiving intravitreal injections, all patients signed an informed consent form. The data collection for this study was carried out between August 13, 2023, and September 10, 2023, at the Department of Ophthalmology, North District, Suzhou Municipal Hospital. We did not have access to personally identifiable participant information during or after data collection; furthermore, the data were anonymized, and the requirement for informed consent was therefore waived.

### 2.2. Study design and patient selection

This was a retrospective observational study in which patients were divided into a treatment-interruption group (TIG) and a treatment-continuous group (TCG). TIG patients with nAMD had to delay anti-VEGF therapy for at least 8 weeks because they were unable to attend their planned visits to Suzhou Municipal Hospital between February 2022 and May 2022, during which the incidence of COVID-19 in the region rapidly increased.

TCG patients were recruited over the same period as nAMD patients who had completed at least three anti-VEGF treatments and received consecutive, timely follow-up anti-VEGF treatment for one year.

#### 2.2.1. Inclusion and exclusion criteria.

The patient inclusion criteria were as follows: 1) >50 years of age, 2) diagnosis of nAMD, 3) ≥3 anti-VEGF intravitreal injections for the active stages of nAMD prior to the treatment delay caused by COVID-19 (via the 3 + PRN protocol), 4) at least 8 weeks of delay in anti-VEGF therapy after the expected follow-up due to COVID-19, 5) 1 year of follow-up after resumption of anti-VEGF therapy, and 6) ≥1 year of anti-VEGF injection after resumption of treatment following the COVID-19–based delay.

The patient exclusion criteria were as follows: 1) no intravitreal anti-VEGF injections for at least one year, 2) a combination of other retinal pathologies in addition to nAMD, 3) refractive media clouding, 4) a history of vitrectomy, and 5) severe underlying systemic disease.

All patients underwent a detailed ophthalmic examination, including BCVA assessment, slit‒lamp examination, intraocular pressure (IOP) examination, fundus examination, optical coherence tomography (OCT), optical coherence tomography angiography (OCTA) and fundus fluorescein angiography (FFA).

For each patient, diagnostic information (date, MNV type, baseline BCVA, and baseline CRT) and the anti-VEGF drugs used were extracted from the medical records.

For the analysis, we used data from the patient’s first visit (baseline), data from the last follow-up visit before treatment interruption (V0), data from the first visit after the COVID-19 lockdown (V1), data from follow-up performed at 6 months (V-6 months) and data from the final visit up to the 1-year follow-up (V-final).

#### 2.2.2. OCT and OCTA imaging.

OCT (Cirrus HD-OCT Model 5000; Carl Zeiss Mediatic, Inc., Dublin, CA, USA) was performed in 512 ×  128 scan mode with a 6 mm ×  6 mm scanning area. A 30° circular scan was performed with the central macular recess as the center of the macula from the nasal to the temporal side. The CRT was defined as the thickness of the retina within 1 mm of the central macular recess.

OCTA (Cirrus HD-OCT Model 5000; Carl Zeiss Mediatic, Inc., Dublin, CA, USA) was performed with 3 × 3 mm and 6 × 6 mm scan patterns. MNV was detected from the en face slab containing the outer retina to the choriocapillaris en face slab. According to the CONAN criteria [18], MNV is defined as type 1, type 2, mixed or type 3. Neovascularizations that was confined to the space under the RPE, as shown by the appearance of vessels below the level of the RPE on OCTA, was defined as type 1 MNV. Neovascular complexes located in the subretinal space above the level of the RPE, as depicted by vascular elements appearing above the level of the RPE on OCTA, were defined as type 2 MNV. OCT findings of both type 1 and type 2 MNV together (that is, neovascularization in the subretinal pigment epithelial and subretinal compartments) were recorded as mixed MNV. Extension of hyperreflectivity from the middle retina toward the level of the RPE associated with intraretinal edema, hemorrhage, and telangiectasis (depicted on OCTA as the downgrowth of new vessels toward or even penetrating the level of the RPE) was defined as type 3 MNV.

Moreover, we evaluated OCTA-derived features of MNV disease activity in patients with nAMD. This included the presence of a feeder vessel, a surrounding hyporeflective halo and the absence of a capillary fringe. Exudative disease activity was assessed on the basis of the presence of active MNV, subretinal fluid (SRF) or intraretinal fluid (IRF).

### 2.3. Outcome measures and statistical analysis

The primary outcome measures were the mean changes in BCVA and CRT, whereas the secondary outcome measure was the change in anatomical features on OCT over the study period.

The BCVA was measured via the international standard visual acuity scale and was converted to the logarithm of the minimum angle of resolution (logMAR) for data analysis.

IBM SPSS Statistics for Windows, version 21 (IBM Corp., Armonk, NY, USA), was used to analyze the data. Continuous variables are described as the means and standard deviations (SDs), whereas other nominal variables are described as counts (frequencies). The McNemar test was used to compare categorical variables. The independent t-test was used for between-group comparisons of the baseline BCVA and CRT, whereas ANOVA was used to compare the BCVA and CRT from baseline to each time point. Factors associated with changes in BCVA were determined with multiple linear regression analysis. A p value <  0.05 was considered to indicate statistical significance.

## 3. Results

### 3.1. Demographic characteristics and clinical outcomes of TIG and TCG patients

**Fig 1:**
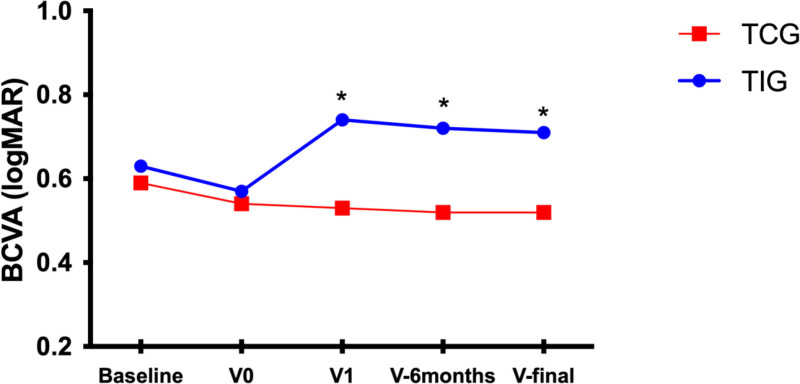
Mean BCVA values over time in TIG and TCG patients. V0: last follow-up visit before treatment interruption; V1: first visit after the COVID-19 lockdown; V-6 months: follow-up visit performed at 6 months; and V-final: the final visit up to the 1-year follow-up. * : significant difference between groups.

**Fig 2:**
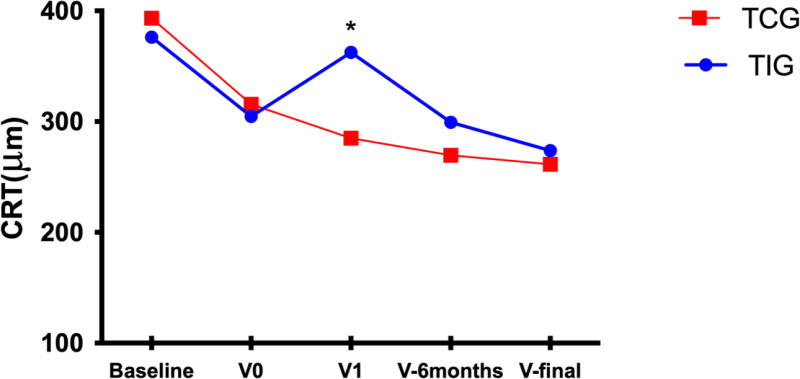
Mean CRT over time between TIG and TCG patients. V0: last follow-up visit before treatment interruption; V1: first visit after the COVID-19 lockdown; V-6 months: follow-up visit performed at 6 months; and V-final: the final visit up to the 1-year follow-up. * : significant difference between groups.

A total of 98 patients (102 eyes) were included in the TIG, and 116 patients (121 eyes) were included in the TCG. The intervals between intravitreal injections were 3.26 ±  1.85 and 1.54 ±  0.44 months for the TIG and TCG, respectively (p <  0.001); the injection intervals for the TIG were delayed by 2.47 ±  1.43 months with respect to those for the TCG. The 2 groups were similar in terms of age (p = 0.925), sex (p = 0.867), lens status (p = 0.788), anti-VEGF drug use (p = 0.816), MNV type (p =  0.805) and treatment duration (p = 0.413). In both groups, the patients presented with a significantly similar mean BCVA (logMAR) (0.63 ± 0.39 in the TIG vs. 0.59 ±  0.31 in the TCG, p = 0.538) and mean CRT (376.20 ± 95.44 µm in the TIG vs. 393.52 ± 102.01 µm in the TCG, p = 0.268) at the start of intravitreal anti-VEGF injections. However, after the COVID-19 lockdown and a subsequent year of treatment, the TIG recovered anatomically to a similar outcome as the TCG did (273.95 ± 112.96 µm in the TIG vs. 261.43 ± 90.66 µm in the TCG, p = 0.322) but experienced a poorer recovery of visual function (0.71 ± 0.38 in the TIG vs. 0.52 ± 0.32 in the TCG, p < 0.001) (Table 1). The mean changes in the BCVA and CRT between the two groups are shown in Figs 1–2.

**Table 1:** Demographic characteristics and clinical outcomes of TIG and TCG patients.

	TIG (n = 98)	TCG (n = 116)	P value
Age, y (mean ± SD)	62.81 ± 7.15	59.4 ± 8.23	0.925
Sex, n (%)			0.867
Male	50	59	
Female	48	57	
Eyes, n (%)	102	121	0.565
MNV type, n (%)			0.805
Type 1	68 (66.7%)	77 (63.6%)	
Type 2	21 (20.6%)	23 (19%)	
Type 3	10 (9.8%)	15 (12.4%)	
Mixed	3 (2.9%)	6 (5%)	
Anti-VEGF drugs, n (%)			0.816
Ranibizumab	76	89	
Aflibercept	5	4	
Conbercept	21	28	
Lens status, n (%)			0.788
Phakic	70	79	
Pseudophakic	32	42	
Anti-VEGF injections at baseline (mean ± SD)	4.31 ± 0.83	4.54 ± 0.95	0.277
BCVA at baseline, logMAR	0.63 ± 0.39	0.59 ± 0.31	0.538
CRT at baseline, µm	376.2 ± 95.44	393.52 ± 102.01	0.268
Intervals between intravitreal injections, months (mean ± SD)	3.26 ± 1.85	1.54 ± 0.44	<0.001^*^
BCVA at V-final, logMAR	0.71 ± 0.38	0.52 ± 0.32	<0.001^*^
CRT at V-final, µm (mean ± SD)	273.95 ± 112.96	261.43 ± 90.66	0.322
Injections at V0 (mean ± SD)	4.83 ± 1.56	--	--
Treatment interruption length, months (mean ± SD)	2.65 ± 0.77	---	--
Treatment duration, y (mean±SD)	2.15 ± 0.95	2.32 ± 1.05	0.452
Anti-VEGF injections at V-final	6.26 ± 2.53	5.17 ± 2.23	0.002^*^

**Note:** V0: last follow-up visit before treatment interruption; V-final: final visit up to the 1-year follow-up. Categorical variables are shown as numbers (%), and continuous variables are presented as the means ±  standard deviations. BCVA, best-corrected visual acuity; logMAR, logarithm of the minimum angle of resolution. CRT, central retinal thickness; anti-VEGF, anti-vascular endothelial growth factor.

*p < 0.05: TIG vs. TCG.

### 3.2. Analysis of visual acuity changes in the TIG

After the COVID-19 lockdown, the mean BCVA in the TIG cohort decreased significantly to 0.74 ± 0.42, which was lower than that in the period before treatment interruption (p = 0.037). After the subsequent year of follow-up, the mean BCVA in the TIG slightly improved to 0.71 ± 0.38; however, compared with that in the period before treatment interruption, the BCVA was significantly worse (p = 0.026) (Table 2).

**Table 2:** Comparison of BCVA values for TIG patients.

	BCVA (logMAR)	vs. V0 (P value)	vs. V-1 (P value)	vs. V-last (P value)
V0	0.57 ± 0.35	0.037^*^	--	0.026^*^
V1	0.74 ± 0.42	--	0.037^*^	0.101
V-6 months	0.72 ± 0.45	0.216	0.029^*^	0.723
V-final	0.71 ± 0.38	0.101	0.026^*^	--

**Note:** V0: last follow-up visit before treatment interruption; V1: first visit after the COVID-19 lockdown; V-6 months: follow-up visit performed at 6 months; and V-final: the final visit up to the 1-year follow-up. The data are presented as the means ±  standard deviations. BCVA, best-corrected visual acuity; logMAR, logarithm of the minimum angle of resolution.

*p < 0.05.

### 3.3. Analysis of visual acuity changes in the TCG

Compared with that at the last visit before the COVID-19 pandemic, the mean BCVA in the TCG cohort improved slightly to 0.53 ± 0.29, and there was no decline; rather, there was an improvement in visual acuity, although there was no significant difference (p = 0.532). At 12 months, the mean BCVA was 0.52 ± 0.35, which was not significantly different from that at the last visit before the pandemic (p = 0.511) (Table 3).

**Table 3:** Comparison of BCVA values for TCG patients.

	BCVA (logMAR)	vs. V1 (P value)
V0	0.54 ± 0.33	--
V1	0.53 ± 0.29	0.532
V-6 months	0.52 ± 0.27	0.499
V-final	0.52 ± 0.35	0.511

**Note:** V0: last follow-up visit before treatment interruption; V1: first visit after the COVID-19 lockdown; V-6 months: follow-up at 6 months; and V-final: the final visit up to the 1-year follow-up. The data are presented as the means ±  standard deviations. BCVA, best-corrected visual acuity; logMAR, logarithm of the minimum angle of resolution. * p < 0.05.

### 3.3. Disease activity assessed by OCT in the TIG

Before treatment interruption, the mean CRT was 304.59 ± 118.72 µm in patients who experienced treatment interruption, although a significant increase in the mean CRT was observed during the treatment interruption period. After a year of standard treatment, the mean CRT decreased and returned to values similar to those observed before the treatment interruption (273.95 ± 112.96 µm at V-final vs. 304.59 ± 118.72 µm at V0, p = 0.152) (Table 4).

**Fig 3:**
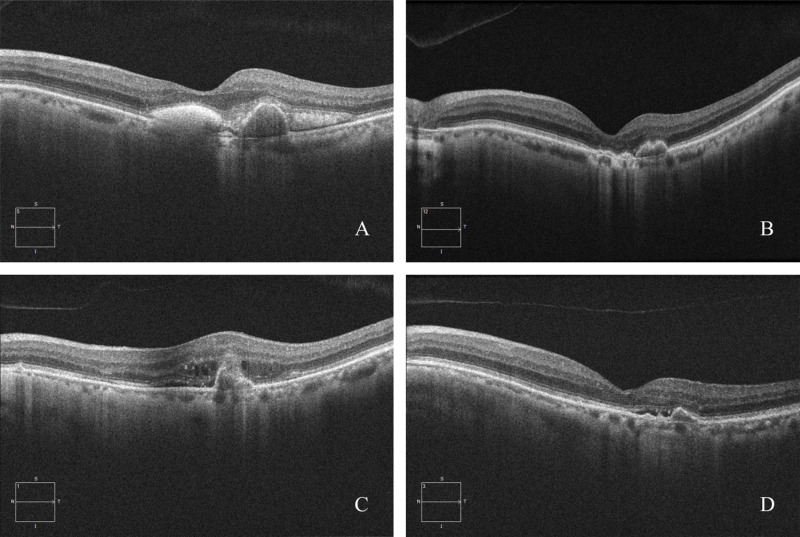
Spectral-domain optical coherence tomography (SD-OCT) images taken from a 72-year-old female patient with neovascular age-related macular degeneration (nAMD) who received ranibizumab injections via a 3 ** + PRN treatment regimen in the right eye prior to COVID-19 lockdown.** The patient’s baseline best-corrected visual acuity (BCVA) was 0.75 (logMAR). OCT image taken at baseline (A) reveals macular neovascularization (MNV), subretinal hyperreflective material (SHRM) and subretinal fluid (SRF). After the first 3 months of treatment, the patient’s BCVA improved to 0.53 (logMAR); the OCT image taken at V0 (B) shows resolution of the subretinal hemorrhage and SHRM. However, the patient’s treatment was interrupted for 3.5 months because of the COVID-19 lockdown. When the patient returned for treatment, the BCVA had decreased to 0.73 (logMAR); the corresponding OCT image taken at V1 (C) shows subretinal hemorrhage, IRF and SRF. After one year of standard treatment, the patient’s BCVA slightly increased to 0.72 (logMAR). The corresponding OCT image taken at V-final (D) shows resolution of the subretinal hemorrhage.

Active disease was defined as the presence of active MNV, SRF or IRF. OCT evaluation revealed active MNV in 66 (64.7%) eyes, SRF in 40 (39.2%) eyes, IRF in 23 (22.5%) eyes, and both SRF and IRF in 16 (15.7%) eyes before treatment interruption. At the end of the COVID-19 lockdown period, the largest increase in the incidence of these conditions was observed in patients with both SRF and IRF, from 15.7% at V0 to 41.2% at V1. During the year of regular follow-up and treatment, the proportion of eyes with both SRF and IRF significantly decreased, returning to a value comparable to that observed before treatment interruption (11 (10.9%) at V-final versus 16 (15.7%) at V0, p = 0.204). Moreover, the percentage of eyes showing SRF on OCT imaging decreased significantly, reaching a level lower than that observed before treatment interruption (22 (21.6%) at V-final versus 40 (39.2%) at V0, p = 0.0017). There was no significant difference in the percentage of eyes presenting with IRF at V-final or V0 (26.6% versus 22.5%, respectively; p = 0.503) (Table 4). Examples of OCT images of an nAMD patient who experienced treatment interruption are shown in Fig 3.

**Table 4:** Comparison of OCT values for TIG patients.

	Active MNV	SRF	IRF	SRF+IRF	CRT (µm)
V0	66 (64.7%)	40 (39.2%)	23 (22.5%)	16 (15.7%)	304.59 ± 118.72
V1	80 (78.6%)	55 (53.9%)	29 (28.4%)	42 (41.2%)	362.62 ± 149.17
V-6 months	44(43.13%)	30(29.41%)	27(26.47%)	22(21.57%)	299.53 ± 155.64
V-final	29 (28.4%)	22 (21.6%)	27 (26.6%)	11 (10.9%)	273.95 ± 112.96
V-final vs. V0 (P value)	0.0015^*^	0.0017^*^	0.503	0.204	0.152
V0 vs. V1 (P value)	<0.001^*^	0.0013^*^	0.499	<0.001^*^	0.041^*^
V-6 months vs. V0 (P value)	<0.001^*^	0.316	0.715	0.253	0.682

**Note:** V0: last follow-up visit before treatment interruption; V1: first visit after the COVID-19 lockdown; V-6 months: follow-up at 6 months; and V-final: the final visit up to the 1-year follow-up. The values are presented as numbers (percentages). MNV: macular neovascularization; CRT: central retinal thickness; SRF: subretinal fluid; IRF: intraretinal fluid.

*p < 0.05.

### 3.4. Multiple linear regression analysis of factors affecting BCVA changes caused by the COVID-19 pandemic

We detected correlations for BCVA changes with age, sex, OCT parameters before treatment interruption, number of injections before treatment interruption, BCVA (logMAR) before treatment interruption, treatment duration, intervals between intravitreal injections and the length of treatment interruption. Multiple linear analysis revealed that longer treatment interruption was associated with deterioration in visual acuity (p = 0.009). The results of the multiple linear regression analysis of the variables influencing BCVA changes are shown in Table 5.

**Table 5:** Multiple linear logistic regression analysis of factors affecting BCVA changes caused by the COVID-19 pandemic.

	Change in BCVA	
	Standardized Beta Coefficient (SE)	P value
Age (years)	-0.116	0.589
Sex	0.030	0.858
Treatment duration (months)	0.429	0.201
the length of treatment interruption	0.415	0.011^*^
Number of injections at V0	-0.507	0.052
Intervals between intravitreal injections	-0.105	0.483
BCVA (logMAR) at V0	1.048	0.220
CRT at V0	-0.158	0.401
SRF at V0	-0.156	0.450
IRF at V0	-0.190	0.260
Active MNV at V0	0.036	0.848
SRF+IRF at V0	0.026	0.901

**Note:** BCVA, best-corrected visual acuity; logMAR, logarithm of the minimum angle of resolution. CRT, central retinal thickness; anti-VEGF, anti-vascular endothelial growth factor. MNV: macular neovascularization; CRT: central retinal thickness; SRF: subretinal fluid; IRF: intraretinal fluid. V0: last follow-up visit before treatment interruption,

*p < 0.05.

### 3.5. Intravitreal Anti-VEGF Treatment

During the year of follow-up, the mean number of anti-VEGF treatments was significantly greater in the TIG group than in the TCG group (6.26 ± 2.53 vs. 5.17 ± 2.23, p = 0.002). After the COVID-19 lockdown, the number of intravitreal injections in the TIG was significantly greater in the first three months than in the other quarters of the year (2.3 versus 1.6, 1.4, and 1.3 in the first, second, third, and fourth quarters, respectively; p = 0.0016).

## 4. Discussion

The COVID-19 pandemic has had a serious impact on healthcare. Maintaining a standardized and adequate regimen is important for anti-VEGF therapy in patients with nAMD. To date, the short-term functional and anatomical outcomes after anti-VEGF therapy delay due to COVID-19 have been widely analyzed [14,19,20]. Here, we analyzed the long-term consequences of delayed anti-VEGF therapy by focusing on the anatomical and functional status of nAMD patients after 1 year of regular follow-up and treatment.

The results of our study revealed that COVID-19 had a significant negative impact on the response to anti-VEGF therapy in ophthalmic nAMD patients at a hospital in southern China, and although the percentage of OCT images with evidence of active disease and the mean CRT were restored to the values observed before the COVID-19 lockdown, visual acuity had not yet reached prelockdown values, suggesting that patients with prolonged therapy interruption are at risk for serious adverse visual sequelae.

Our findings are similar to those of Greenlee et al. [21]. In addition, Elfalah et al. [11] and Naravane et al. [12] compared the BCVA and CRT in patients with delayed intravitreal injections in different disease groups due to COVID-19. In both studies, nAMD patients experienced BCVA deterioration after treatment delay, but the CRT decreased in one study and increased in another. The difference in the results between these two studies may be related to differences in the delay intervals.

Recent studies have shown that IRF, SRF, PED and SHRM are important indicators of nAMD activity, especially IRF and SRF [22,23]. A multicenter study in Spain reported a 3.1% increase in the incidence of active disease (SRF and IRF) on OCT imaging 6 months after the start of the COVID-19 lockdown [24]. However, Yeter et al. [7] demonstrated that during a mean of 3.5 ± 1 months of follow-up after the COVID-19 lockdown, the OCT findings (SRF, IRF, and SRHM) returned to values observed at prelockdown visits. Kato et al [25]. found an increase in the percentage of OCT findings (SRF, IRF, and SRHM) after 6 months of follow-up after the COVID-19 epidemic and argued that the presence of SRF at baseline was negatively correlated with the deterioration in maximum SRF height. Nevertheless, the variability in the results of these studies may be due to differences in follow-up durations (Kato et al. performed their follow-up 6 months after the lockdown; Yeter et al. had a follow-up of 3.5 ± 1 months; and the Spanish multicenter study performed the follow-up 6 months after the lockdown).

Our study has several limitations. Given the retrospective nature of the study, selection bias is expected. Furthermore, our small study population and the use of logMAR visual acuity scores rather than ETDRS letter scores both limited the power of the analysis.

## 5. Conclusions

Our results revealed that the review of some patients was delayed by 8 weeks or more due to the COVID-19 pandemic lockdown, and these patients were greatly affected by the delayed review, accompanied by loss of vision and worsening of OCT scanning activity. Despite restarting anti-VEGF therapy, this vision loss persisted during follow-up. The unfortunate situation experienced by these patients demonstrates that temporary discontinuation of anti-VEGF therapy for nAMD has functional consequences and that these consequences are not fully reversible after 12 months of standard therapy. While anatomical deterioration appears to be somewhat reversible with regular intravitreal treatment, the damage already done to the retina appears to prevent vision from reaching previous levels.

Our findings are useful for predicting the outcomes of patients who experience unplanned interruptions in intravitreal anti-VEGF injections and for providing suggestions for those patients to decide whether to continue treatment while weighing the risk of the pandemic.

Future studies with long-term longitudinal follow-up of this population may provide more substantial information. Importantly, these studies may elucidate whether the COVID-19 pandemic has negatively affected long-term outcomes.
